# Proinflammatory activation profile in circulating monocytes in patients with a major depressive episode

**DOI:** 10.1192/j.eurpsy.2023.2112

**Published:** 2023-07-19

**Authors:** L. Grendas, R. Alvarez Casiani, A. Arena, M. Penna, F. Hunter, A. Olaviaga, C. Prokopez, V. Tifner, A. Armesto, A. Carrera Silva, A. Errasti, F. Daray

**Affiliations:** 1 Institute of Pharmacology, School of Medicine, University of Buenos Aires; 2 Institute of Experimental Medicine, IMEX-CONICET- National Academy of Medicine, Buenos Aires, Argentina

## Abstract

**Introduction:**

Mood Disorder (MD) affects more than 300 million people globally, and its etiology is unknown. In recently published data, MD has been correlated with inflammation and the immune system. Circulating monocytes have been proposed to play a role in the pathophysiology of depression.

**Objectives:**

To determine if there is a specific activation profile of monocytes in patients with MD that differentiates them from healthy control (HC).

**Methods:**

Study Design: Case-control study matched by sex and age. The study was approved by IRB and carried out in three hospitals in Argentina.Participants between 18 and 55 years old from both genders, were evaluated by psychiatrists using the International Psychiatry Interview (MINI) to diagnose Mood Disorder (MD), and the Hamilton Depression Rating Scale (HADRS) to define active disease (AD), non-active disease (NAD) or healthy control (HC). The three monocyte subtypes were directly stained and analyzed in a drop of 100 uL of blood sample based on our validated monocyte cocktail including CD11b, HLA-DR, CD86, CD14 and CD16 expression by flow cytometry. To define normality Kolmogorov-Smirnov test was employed. A parametric T-test with Welch´s correction was employed for normal distribution and a non-parametric Mann Whitney test was used when comparing populations that do not pass the normality test.

**Results:**

The sample characteristics were shown in Table 1. Patients with AD (Hamilton >7) (n: 37), patients with NAD (Hamilton <7) (n: 38), and HC (n: 39) were recruited. The percentage of classical monocytes decreased in AD vs NAD (p=0.04), both AD, and NAD have significantly lower levels of classical monocytes than HC (****p<0.001) (Image 1). The percentage of intermediate monocytes is higher in AD vs NAD (p=0.05), both AD, and NAD have significantly higher levels of intermediate monocytes than HC (****p<0.001) (Image 2). The percentage of non-classical monocytes is higher in AD vs NAD (p=0.05), both AD, and NAD have significantly higher levels of non-classical monocytes than HC (****p<0.001) (Image 3).

**Table 1.** General characteristics of the sample

	Active disease	Non-active disease	Healty control
n	37	38	39
Age (SD)	42.95 (11.78)	42 (12.02)	40.67 (11.42)
Women (%)	76.3	64.9	76.9
BD I	15.8	54.1	0.0
BD II	26.3	5.4	0.0
BD (non specified)	0.0	2.7	0.0
MDD	57.9	37.8	0.0
HAM-D 17 items mean (SD)	14.13 (4.89)	3.11 (2.35)	0.49 (0.85)

**Image:**

**Figure:**
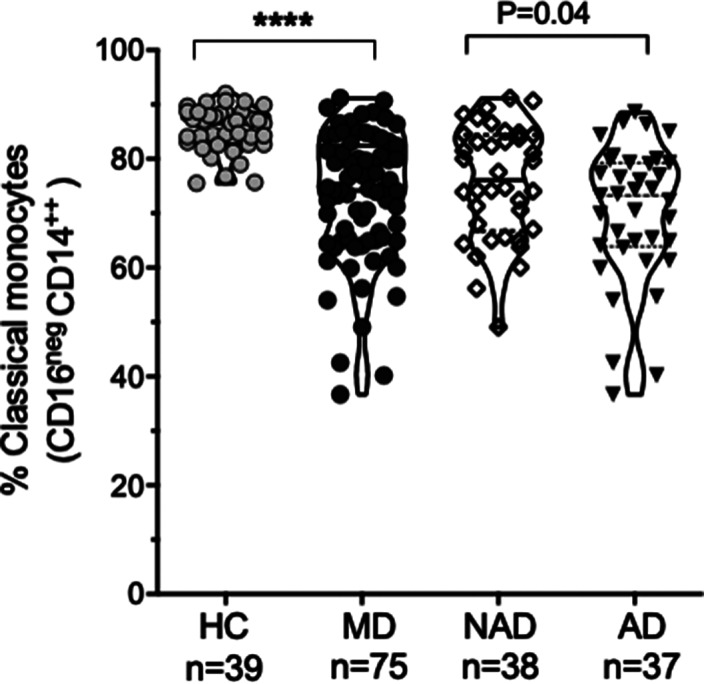

**Image 2:**

**Figure:**
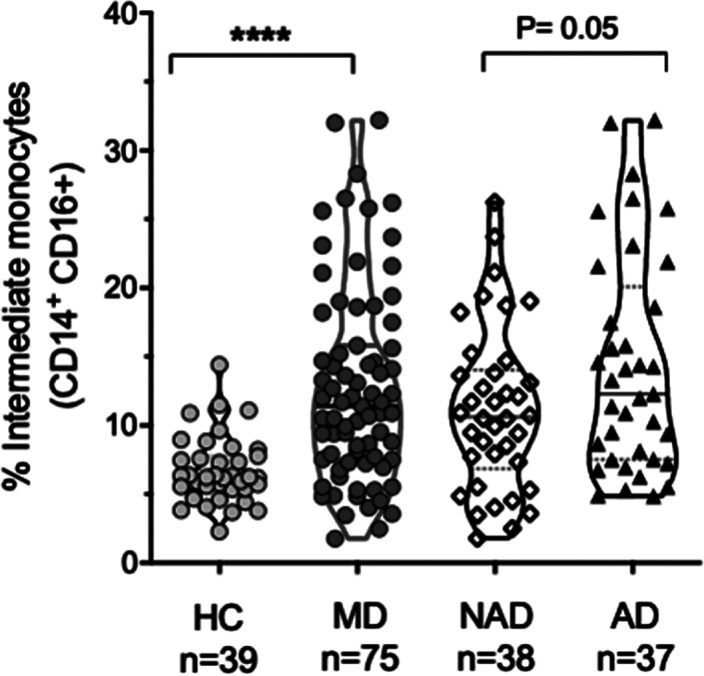

**Image 3:**

**Figure:**
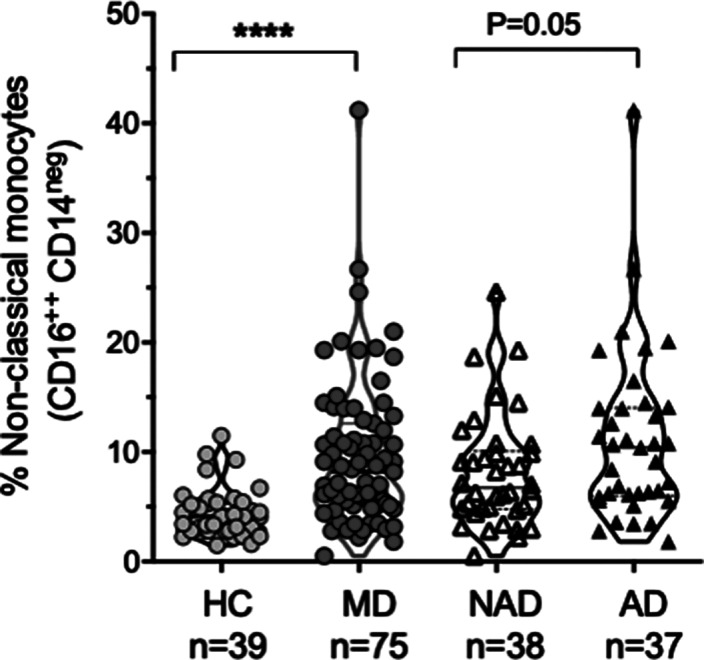

**Conclusions:**

While comparing percentages of three different monocyte subsets, clear differences in their distribution among the control and patient groups were appreciated. After comparing the subset frequencies between active patients (AD) and patients who were in remission (NAD), significant differences among the subsets were found although without reaching values of the HC, indicating that even patients in remission show an activated monocyte profile.

**Disclosure of Interest:**

None Declared

